# Exposure to an environmental estrogen breaks down sexual isolation between native and invasive species

**DOI:** 10.1111/j.1752-4571.2012.00283.x

**Published:** 2012-07-10

**Authors:** Jessica L Ward, Michael J Blum

**Affiliations:** 1 Department of Ecology and Evolutionary Biology, Tulane UniversityNew Orleans, LA, USA; 2 Department of Fisheries, Wildlife and Conservation Biology and Department of Ecology, Evolution and Behavior, University of MinnesotaSaint Paul, MN, USA

**Keywords:** bisphenol A, hybridization, mate choice, environmental change, fish, xenoestrogen, communication, visual signals

## Abstract

Environmental change can increase the likelihood of interspecific hybridization by altering properties of mate recognition and discrimination between sympatric congeners. We examined how exposure to an environmentally widespread endocrine-disrupting chemical (EDC), bisphenol A (BPA), affected visual communication signals and behavioral isolation between an introduced freshwater fish and a native congener (genus: *Cyprinella*). Exposure to BPA induced changes in the expression of male secondary traits as well as male and female mate choice, leading to an overall reduction in prezygotic isolation between congeners. Changes in female mate discrimination were not tightly linked to changes in male phenotypic traits, suggesting that EDC exposure may alter female choice thresholds independently of the effects of exposure on males. These findings indicate that environmental exposure to EDCs can lead to population declines via the erosion of species boundaries and by promoting the establishment and spread of non-native species via hybridization.

## Introduction

Environmental degradation can alter evolutionary processes responsible for the origins and maintenance of biodiversity. Anthropogenic environmental changes that promote interspecific hybridization between sympatric species are of particular concern (Taylor et al. 2006; Seehausen et al. 2008), especially for fishes and other groups where prezygotic reproductive barriers are more prevalent than postzygotic barriers to genetic exchange (Mendelson 2003). Interspecific differences in phenotypic traits that serve as mate recognition signals (e.g., shape, color pattern, behavior) can maintain reproductive isolation between closely related species (Ptacek 2000), but environmental degradation can alter the expression, transmission, and reception of these signals (Seehausen et al. 1997; Fisher et al. 2006; van der Sluijs et al. 2010). For example, eutrophication and elevated sediment loads can weaken intraspecific sexual selection upon male traits and/or disrupt interspecific mate choice in fishes by decreasing the conspicuousness of male visual signals and compromising the discriminatory ability of females (Seehausen et al. 1997; Candolin et al. 2007). Similar outcomes can occur in response to anthropogenic input of chemical contaminants that alter the expression of signals used in mate choice or that induce endogenous changes in receiver response (Fisher et al. 2006; Saaristo et al. 2010).

Rivers and streams are frequent environmental endpoints for pharmaceuticals and compounds used in the manufacture of household products (e.g., 17α-ethinyl estradiol, bisphenol A, nonylphenol, octylphenol) that disrupt endocrine signaling pathways (Kolpin et al. 2002). In fishes, endocrine-disrupting chemicals (EDCs) can alter reproductive behavior and physiology by mimicking the action of natural hormones (Arukwe 2001; Mills and Chichester 2005). In addition to regulating gonadal function and the expression of sexually selected courtship traits in males (Borg 1994; Mayer et al. 2004), hormones can mediate aspects of sex and species recognition (Thompson et al. 2004; Gabor and Grober 2010) and modulate variation in female spawning receptivity and mate permissiveness (Clement et al. 2005; Ramsey et al. 2011; see also Lynch et al. 2006). As a consequence, EDC exposure not only can influence individual reproductive success and population viability (Jobling and Tyler 2003; Kidd et al. 2007) but may also influence reproductive isolation between species.

Exposure to EDCs can modify the strength or form of natural and sexual selection (e.g., stabilizing, directional) in populations. Intraspecific mate choice experiments have shown that exposure to endocrine-disrupting chemicals can disrupt social dominance hierarchies and the competitive ability of individuals (Kristensen et al. 2005; Coe et al. 2008; Saaristo et al. 2009a), relax sexual selection operating on male traits (Saaristo et al. 2009b), and induce changes in the expression of male visual signals that females use for mate recognition and discrimination (Bayley et al. 1999; Bjerselius et al. 2001; Saaristo et al. 2010). However, how these changes influence interspecific reproductive interactions is unknown. Exposure-induced behavioral or phenotypic changes could reduce or eliminate behavioral isolation between sympatric species in affected environments if females discriminate against altered cues in conspecific males (Arellano-Aguilar and Garcia 2008; Saaristo et al. 2009a; Partridge et al. 2010) or if the ability to distinguish between males is compromised (Fisher et al. 2006; Saaristo et al. 2009b; Shenoy et al. 2010). Modified thresholds of male and female mate discrimination (e.g., increased permissiveness) resulting from EDC exposure could also increase the likelihood of hybridization independently of changes in visual signals.

In this study, we examine how short-term exposure to bisphenol A (BPA), an environmentally widespread xenoestrogenic compound that is used in the manufacture of plastics and resins (Crain et al. 2007), affects visual communication and prezygotic reproductive isolation within the broader context of biological invasions involving hybridization in stream fishes. We test for evidence of male and female assortative mate choice between introduced red shiner (*Cyprinella lutrensis*) and native blacktail shiner (*C. venusta*) under control conditions and following short-term (14 days) exposure to BPA. We compare the overall degree of prezygotic reproductive isolation between treatment groups and examine sex- and species-specific contributions to behavioral isolation within and between treatment groups. In addition, we quantify the effects of EDC exposure on male secondary sexual characteristics that serve as visual signals during reproduction and examine the relationships between male traits and female mate choice in control and exposed fish. This approach enabled us to determine whether changes in female interspecific mate assessment based on individual visual signals correspond to changes in male signals resulting from EDC exposure.

## Materials and methods

### Study system

*Cyprinella venusta* and *C. lutrensis* are crevice-spawning species that aggregate in single-species and mixed-species groups during the breeding season. Males generally aggregate over spawning substrate and engage in aggressive male–male interactions. Females tend to remain separate until inclined to spawn, when females will approach males to initiate breeding (Minckley 1972). Upon initiation, a male will court a female by circling and leading them to a spawning site (rocks, twigs, etc.) where the female will deposit her eggs. The male quickly fertilizes the eggs after which the spawning partners separate (Minckley 1972; Gale 1986).

Hybridization between *C. lutrensis* and its congeners has been well studied (Walters et al. 2008; Blum et al. 2010; Broughton et al. 2011; Ward et al. 2012), including episodes linked to species introductions and environmental change (Hubbs and Strawn 1956; Page and Smith 1970; Walters et al. 2008; Ward et al. 2012). One of the best-studied species interactions is between introduced *C. lutrensis* and native *C. venusta* in the Upper Coosa River Basin (Alabama, Georgia, and Tennessee, USA). Morphological and genetic assays of hybridization have shown that *C. lutrensis* × *C. venusta* hybrids can dominate in some mainstem reaches (Ward et al. 2012) and that the presence of hybrids in tributaries corresponds to the presence of native *C. venusta* as well as industrial and agricultural land use (Walters et al. 2008). Controlled experiments have additionally shown that prezygotic isolation between *C. lutrensis* and *C. venusta* is stronger than postzygotic isolation (Blum et al. 2010).

### Collection, maintenance, and care

We collected adult *C. lutrensis* and *C. venusta* via seining in April and May 2010 from Proctor Creek, GA, USA (33.795, −84.475), and Sugar Creek, GA, USA (34.920, −84.842), respectively. Site selection followed the analysis of genetically based morphological traits, mitochondrial markers, and nuclear loci, indicating that the populations exhibited no signs of hybridization (Walters et al. 2008). We transported wild-caught fish to the laboratory in aerated containment units, where they were permitted to acclimate to laboratory conditions for 4 weeks in mixed-sex, single-species, 378-L opaque polyethylene tubs equipped with spawning towers and a continuous flow-through of filtered and UV-sterilized water. One week prior to experimentation, we transferred male and female *C. venusta* and *C. lutrensis* from the holding tubs to 75-L glass tanks where they were maintained in low-density (6–8 *C. venusta*, 10–14 *C. lutrensis*), mixed-sex (equal numbers of males and females), single-species communities to promote reproductive behavior and ease subsequent introduction to glass-walled exposure tanks. Community tanks were visually isolated from one another by the use of opaque dividers. Ambient summer conditions (16 h:8 h light/dark regime, 23–25°C) were maintained throughout the duration of the experiment. Individuals received premium tropical flake food three times daily.

### Exposure regime

We examined communication and mate choice within three treatments: BPA, solvent control (Control_solvent_), and water control (Control_H2O_). Reproductively motivated male and female *C. lutrensis* and *C. venusta* were selected from the community tanks on the basis of sexually dimorphic phenotypic traits, including male breeding coloration and body shape (Page and Burr 1991). At the start of the experiment, *C. lutrensis* and *C. venusta* allocated to Control_solvent_, Control_H2O_, and BPA treatment groups were phenotypically comparable within species and sex classes (see Supporting Information). Individuals in the BPA and Control_solvent_ treatments were allocated to 38-L glass tanks containing carbon-filtered water treated with either 1280 μg L^−1^ BPA (BPA treatment group, Sohoni et al. 2001; Mandich et al. 2007) dissolved in triethylene glycol solvent or an equivalent volumetric percentage of solvent (Control_solvent_ group, 0.00002% by volume). Triethylene glycol is a straight-chain dihydric aliphatic alcohol that has been used as a solvent in similar exposure studies (Cripe et al. 2009). Four conspecific fish (two male and two female) were housed in each exposure tank, and all aquaria were visually and chemically isolated from each other throughout the exposure period. Aeration was provided by the addition of airstones suspended from silicon surgical tubing. We maintained these fish for 14 days under a static daily renewal protocol (Partridge et al. 2010). Tanks were drained and replaced with freshly treated water every 24 h (well within the 4.5-day environmental half-life of BPA, Cousins et al. 2002) and were scrubbed every 48 h to remove debris. Water quality was tested at regular intervals throughout the exposure period (NO_2_ = 0–0.2 ppm, NH_3_/NH_4_ = undetectable, pH = 7.8–8.4).

We maintained individuals allocated to the Control_H2O_ treatment in 378-L laboratory stock tubs equipped with the continuous flow of filtered and UV-sterilized water for the duration of the experiment, which is more representative of natural breeding conditions (Minckley 1972). We compared the behavior of individuals in the two control treatments (Control_solvent_ and Control_H2O_) to determine whether male and female baseline responses were affected either by the static exposure experimental setup regime or by exposure to the solvent (see Supporting Information).

### Behavioral assays

We conducted behavioral trials on the 15th day of the experiment (Mandich et al. 2007). Behavioral responses of female and male *C. lutrensis* and *C. venusta* were examined using dual-choice mate choice assays. In total, we conducted six sets of mate choice trials (*n* = 20 for each set, 120 individual trials), representing all possible combinations of male and female *C. lutrensis* and *C*. *venusta* within each of the bisphenol A, Control_solvent_, and Control_H2O_ treatments (see Table S1). Presumably, all individuals are similarly exposed to EDCs in affected environments. Thus, focal trios used in each trial were selected according to treatment to most accurately represent natural scenarios (i.e., within the same treatment group). We conducted experimental trials in 208-L aquaria divided into three chemically isolated compartments by clear Plexiglas barriers and covered on the back and sides with brown paper. Trials were undertaken in tanks filled with filtered water free from both solvent and BPA. Aquaria were drained and replaced with fresh water prior to each trial. A spawning tower was placed against the back wall of each distal chamber, and illumination was provided by two 15-W full-spectrum bulbs suspended 10 cm above each tank. Experimental males were introduced into the distal compartments of the test tanks and permitted to acclimate for one hour. During acclimation, the opposing males were chemically and visually isolated from the central compartment and from each other via removable, black, opaque dividers fitted over the clear Plexiglas barriers. All individuals remained chemically isolated from one another once the opaque barriers were removed and throughout the duration of the trial.

We conducted trials following Ward and McLennan (2009). A female was introduced into the central compartment of a test aquarium and allowed to acclimate to her surroundings for 10 min. Following acclimation, the female was presented with the stimulus males via the removal of the opaque dividers. We filmed interactions between the female and both males for 10 min from behind a blind. For scoring purposes, the female compartment of the experimental tank was divided into three 18.5-cm zones. The two zones that were closest to the flanking stimulus males were designated as ‘preference zones,’ and the central zone was designated as a ‘neutral zone.’ Interactions between males and females were recorded only within the preference zones. To control for the effects of familiarity, females were not presented with males with whom they had previously shared a tank. Following trials, male and female participants were measured for standard length from the tip of the snout to the rounded edge of the caudal peduncle using digital calipers calibrated to 0.01 mm precision. Males were also scored for color intensity.

We used twenty sets of paired *C. lutrensis* and *C. venusta* males for each experimental series. One trial was eventually discarded in the BPA exposure series because of technical failure [Control_solvent_ (*n* = 20); Control_H2O_ (*n* = 20); BPA (*n* = 19)]. To control for heterogeneity across male pairs, we tested one female of each species with each set of males (*C. lutrensis* females: *n* = 20 in each treatment except for BPA, where *n* = 19; *C. venusta* females: *n* = 20 in each treatment except for BPA, where *n* = 19; see Table S1). Males were permitted a 1-h rest period between female presentations, and trial sequences were balanced with respect to the order of female species presentation, as well as the relative flanking positions of *C. lutrensis* and *C. venusta* males (i.e., to the left or right of the female compartment).

### Female behavior

We determined female mate preference on the basis of side association (time spent in each preference zone) and the number of times that females entered the preference zones of both males. Female behavior in all trial series satisfied parametric assumptions of normality and homogeneity of variance. We tested for assortative female mate choice within each trial series via Bonferroni-corrected *t*-tests conducted upon female responses to paired conspecific and heterospecific males.

### Male behavior and phenotypic variation

#### Courtship

We quantified male courtship toward females of both species by the amount of time that males spent interacting with females, defined as physical contact between a male's snout and the glass divider, and by the frequency with which males initiated bouts of courtship interaction. Behavioral variables were generally normally distributed and satisfied parametric assumptions of homogeneity of variance. We tested for male assortative mate choice within individual trial series via Bonferroni-corrected *t*-tests conducted upon male responses toward sequentially presented conspecific and heterospecific females. We tested for interspecific and between-treatment variation in the total amount of male courtship activity performed via multivariate analysis of variance (manova).

#### Color

To examine broad-scale differences in the intensity of coloration of live males, we adopted a scaled intensity scoring method that is often used in mate choice studies involving stream fishes, including shiners (e.g., Casalini et al. 2009; Walters et al. 2008; Ward and McLennan 2009; Kozak et al. 2011 and references therein; Ward et al. 2012).

##### Cyprinella lutrensis

Breeding male *C. lutrensis* express intense, sexually dimorphic red fin and head coloration and iridescent blue dorsolateral body color (Page and Burr 1991). Prior to exposure (Control_solvent_, BPA) and following each trial (all treatments), one researcher (JLW) assigned live males' individual color intensity values by eye ranging from zero (least intense) to five (most intense) over five morphological regions of the body by comparing fin (caudal, anal, pelvic), head, and body hue with red and blue commercial color standards consisting of six linearly arranged, equally varying color saturations of the appropriate hue (Sherwin-Williams, Cleveland, OH, USA). The intensities of fin color scores were subsequently summed to produce an overall fin score ranging between 0 and 15. Total male color intensity scores were calculated as the sum of scores over all five body regions (head+fins+body: range = 0–25).

We tested for between-treatment (Control_H2O_, Control_solvent_, BPA) differences in the intensities of male color following behavioral trials using manova. Preliminary screening indicated that total male color intensity scores satisfied parametric assumptions of normality [1-sample Kolmogorov–Smirnov test (Control_H2O_: *Z* = 0.71, *P* = 0.71; Control_solvent_: *Z* = 0.65, *P =* 0.80; BPA: *Z* = 0.74, *P =* 0.65)] and homogeneity of error variance (Levene's test: *F*_2,56_ = 0.36, *P* = 0.70). In addition, we examined the extent of phenotypic change over the exposure period within Control_solvent_ and BPA treatments using paired *t*-tests to compare color scores recorded prior to introduction to the exposure tanks and following the 14-day exposure period. Because males were not individually marked upon introduction to the exposure holding tanks (i.e., two males per tank), pre-exposure and post-exposure male color scores were independently averaged across both males within each exposure tank (Control_solvent_: *n* = 12 exposure tanks; BPA: *n* = 16 exposure tanks).

##### Cyprinella venusta

Male and female *C. venusta* are characterized by the presence of a non-sexually dimorphic black caudal spot. Although the caudal spot is unlikely to be affected by fluctuating changes in hormone levels, we scored the intensity of the melanic caudal spot of male *C. venusta* on a scale from zero to three. A score of zero represented no color (not observed) and three represented an intensely black spot (Walters et al. 2008; Ward et al. 2012). Where appropriate, we compared the extent of phenotypic variation between treatments, and across the exposure period, using the same statistical methods described for *C. lutrensis*.

### Sexual isolation

To examine the effects of exposure on the overall strength of prezygotic reproductive isolation between the species, we calculated the strength of conspecific discrimination (*q*_x_) for each female (time spent near = *q*_*time*_, frequency of approach = *q*_*approach*_) and male (time spent engaged in courtship behavior = *q*_*court time*_, number of courtship bouts = *q*_*court bouts*_) behavioral measure according to the equation:


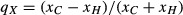
(1)

where *x* is the measured response and subscripts *C* and *H* are conspecific and heterospecific mates, respectively (Stelkens and Seehausen 2009; Ward and McLennan 2009). In this case, −1 represents complete discrimination in favor of the heterospecific mate, and 1 represents complete discrimination in favor of the conspecific mate. This approach allowed us to combine and compare behavioral responses that differed with respect to sex and measurement unit. Preliminary analysis indicated that, across treatments, individual measures of conspecific discrimination were highly correlated within males and females, respectively [males (*q*_*court time*_, *q*_*court bouts*_: Pearson *r* = 0.88, *P* < 0.001); females (*q*_*time*_*, q*_*approach*_: Pearson *r* = 0.86, *P* < 0.001)]. Individual measures (*q*_x_) were therefore averaged to generate a single measure of behavioral isolation (*I*) for each male and female in each trial. We conducted a nested anova with treatment (Control_solvent_, Control_H2O_, BPA) and species origin (*C. lutrensis*, *C. venusta*) specified as fixed effects and included the treatment × species interaction term. We also included sex (nested within the treatment × species interaction term) in the model to examine whether males and females within species differed in strength of conspecific discrimination.

### Female responses to male traits

To determine what male traits females responded to and whether females altered assessment strategies based on individual male traits between treatments, we examined the relationships between the strengths of female response to individual males and hormonally influenced male phenotypic traits (male color, courtship behavior) using general linear models. We modified eqn (1) to calculate the strengths of female responses toward each male in each trial based on association time and frequency of approach and averaged the resulting values across both measures for each female. Analyses examining female responses to *C. lutrensis* and *C. venusta* males, respectively, were conducted separately with treatment (Control_H2O_, Control_solvent_, BPA) and female species (*C. lutrensis, C. venusta*) specified as fixed factors and male traits specified as covariates. For both models, we used principal components to derive an overall courtship score for each male in each trial based on the intensity of sexual displays (time spent courting the female and number of courtship bouts). Male *C. lutrensis* body color intensity (head+fins+body) was included in the appropriate model. Male *C. venusta* coloration did not vary within or across treatments (see Results) and was excluded from analysis. All main effects and trait × main effect interactions were included. Using this approach, significant interaction terms could be interpreted as evidence of variation across treatments in the strength of female responses to individual male traits.

## Results

### Reproductive isolation

#### Female mate choice

**Figure 1:**
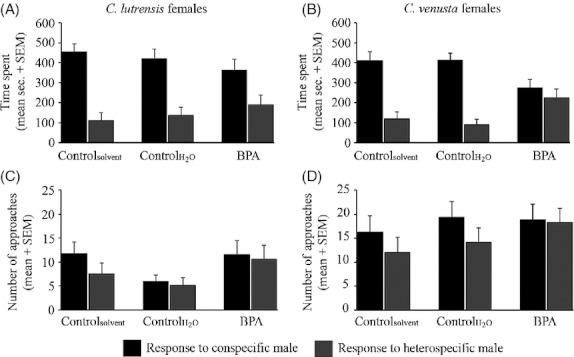
Behavioral responses of female *Cyprinella lutrensis* and female *C. venusta* toward simultaneously presented conspecific and heterospecific males in Control_H2O_, Control_solvent_, and BPA treatments.

Multivariate analysis of variance conducted upon the total amount of time that females spent associating with available males and the total frequency of male visits indicated that levels of reproductive motivation did not differ between treatments for females of either species (*C. lutrensis* females: *F*_4,112_ = 1.80, *P* = 0.13; *C. venusta* females: *F*_4,112_ = 0.47; *P* = 0.76). However, female assortative mate choice differed with respect to exposure regime. Females in both Control_solvent_ and Control_H2O_ treatments discriminated in favor of conspecific mates. Control *C. lutrensis* and *C. venusta* females spent significantly more time associating with conspecific males than heterospecific males (Table 1, Fig. 1A,B) and approached conspecific males more frequently (Table 1, Fig. 1C,D). In contrast, BPA-treated females failed to discriminate between conspecific and heterospecific males on the basis of either measure.

**Table 1:** Behavioral responses [mean (SEM)] of control (H_2_O, solvent; *n* = 20) and exposed (BPA; *n* = 19) male and female *Cyprinella lutrensis* and *C. venusta* toward potential conspecific and heterospecific mates. See text for variable descriptions

Treatment	Control_H2O_				Control_solvent_				BPA			

			Paired *t*-test				Paired *t*-test				Paired *t*-test	
									
Male/Female	Consp.	Heterosp.	*t*_19_	*P*	Consp.	Heterosp.	*t*_19_	*P*	Consp.	Heterosp.	*t*_18_	*P*
Female responses												
*C. lutrensis*												
Time spent (s)	419.58 (48.27)	137.45 (41.59)	3.19	**0.005**	453.99 (41.80)	110.10 (39.37)	4.26	**<0.001**	362.12 (54.17)	189.82 (49.99)	1.67	0.11
Approaches (number)	5.95 (1.37)	5.10 (1.67)	0.66	0.52	11.75 (2.35)	7.50 (2.30)	2.44	**0.03**	11.53 (2.85)	10.58 (2.89)	0.42	0.68
*C. venusta*												
Time spent (s)	411.93 (37.49)	91.19 (24.95)	5.25	**<0.001**	410.66 (42.78)	119.45 (35.03)	3.82	**0.001**	274.60 (41.21)	225.74 (42.37)	0.60	0.55
Approaches (number)	19.30 (3.26)	14.15 (2.99)	2.43	**0.03**	16.20 (3.42)	12.05 (3.05)	1.70	0.11	18.84 (3.22)	18.26 (2.96)	0.25	0.81
Male responses												
*C. lutrensis*												
Courtship time (s)	201.56 (39.13)	38.44 (16.30)	4.47	**<0.001**	208.62 (41.39)	43.45 (17.58)	3.92	**0.001**	155.57 (39.09)	110.01 (32.60)	0.89	0.38
Courtship bouts (number)	35.90 (6.81)	11.35 (3.34)	3.20	**0.005**	35.70 (8.71)	13.25 (5.46)	2.20	**0.04**	25.84 (5.78)	21.05 (4.18)	0.91	0.38
*C. venusta*												
Courtship time (s)	111.14 (27.81)	49.54 (27.40)	1.62	0.12	78.06 (22.65)	18.83 (10.21)	2.63	**0.02**	72.50 (21.03)	30.52 (11.38)	1.61	0.13
Courtship bouts (number)	28.50 (6.08)	7.25 (2.75)	3.64	**0.002**	22.55 (5.57)	8.75 (4.31)	2.75	**0.01**	16.84 (4.36)	8.05 (2.45)	1.72	0.10

Significant values given in bold

#### Male mate choice

**Figure 2:**
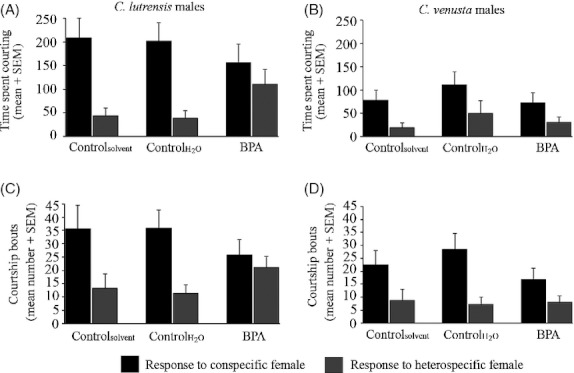
Behavioral responses of male *Cyprinella lutrensis* and male *C. venusta* toward sequentially presented conspecific and heterospecific females in Control_H2O_, Control_solvent_, and BPA treatments.

Male responses toward conspecific and heterospecific mates mirrored the results obtained for females in the three treatments. Control_solvent_ and Control_H2O_ males favored conspecific females; *C. lutrensis* and *C. venusta* males within control treatments spent more time engaged in courtship with conspecific females (Table 1, Fig. 2A,B) and also initiated more bouts of courtship when presented with conspecific mates (Table 1, Fig. 2C,D). In contrast, males within the BPA treatment failed to discriminate between conspecific and heterospecific females on the basis of either measure.

#### Behavioral species isolation

**Figure 3:**
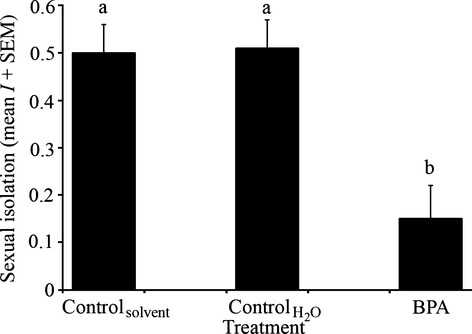
Differences in the strength of sexual isolation (*I* ) between *Cyprinella lutrensis* and *C. venusta* between Control_solvent_, Control_H2O_ (*n* = 20, respectively), and BPA treatments (*n* = 19). Values represent the mean strength of behavioral isolation (+ SEM) averaged over all individuals within trial series. Letters (a,b) represent significantly weaker sexual isolation (*I*) between species exposed to BPA compared to control treatments. Differences are significant at α = 0.001.

The nested anova examining the effects of treatment (Control_solvent_, Control_H2O_, BPA), species (*C. lutrensis, C. venusta*), and sex on variation in sexual isolation (*I*) revealed a significant overall effect of treatment on the strength of prezygotic species isolation (*F*_2,224_ = 9.95, *P* < 0.001, Fig. 3); subsequent pairwise *post hoc* tests indicated that the strength of behavioral isolation between species exposed to BPA was significantly weaker than the strength of behavioral isolation exhibited under control conditions (Control_solvent_: *P* < 0.001, Control_H2O_: *P* < 0.001). No species asymmetries in the degree of sexual isolation were found (species effect: *F*_1,224_ = 0.11, *P* = 0.74), and the two species did not respond differentially to BPA (species × treatment interaction: *F*_2,224_ = 0.16, *P* = 0.85). In addition, we did not detect statistically significant sex-specific asymmetries in the strength of conspecific discrimination for either species within any treatment group (*F*_6,224_ = 0.47, *P* = 0.83).

### Male phenotypic variation and female responses to male traits

#### Effects of exposure on male color

##### Cyprinella lutrensis

**Figure 4:**
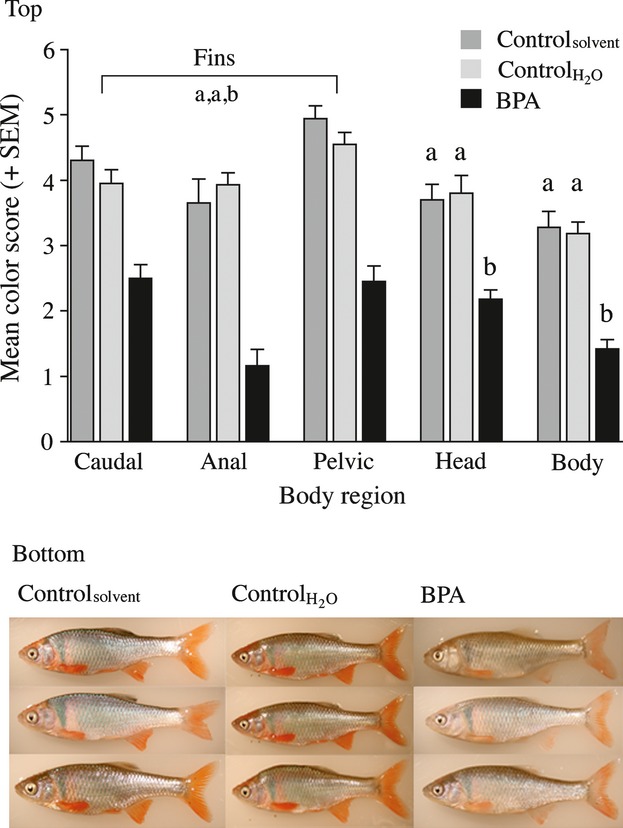
Intensity of male *Cyprinella lutrensis* body color in control and BPA treatments. (Top) Mean intensity scores (+ SEM) of male *C. lutrensis* body coloration within BPA (*n* = 19), Control_solvent_, and Control_H2O_ treatment groups (*n* = 20, respectively). Color was recorded for individual males over five morphological body regions immediately following mate choice trials; fin color scores were summed prior to statistical analysis. Letters (a,b) indicate significantly lower scores recorded for BPA treatment males than for males in either control group (fins, head, body: all *P* < 0.001). (Bottom) Photographs of representative male *C. lutrensis* demonstrating variation in the intensity of fin, head, and dorsolateral body coloration between BPA and control treatments; all photographs were taken immediately following mate choice trials.

Post-trial (day 15) color scores of individual *C. lutrensis* males differed significantly between treatments [fins (*F*_2,56_ = 39.55, *P* < 0.001), head (*F*_2,56_ = 14.66, *P* < 0.001), and body (*F*_2,56_ = 28.01, *P* < 0.001)]. According to *post hoc* tests, mean color scores of BPA-treated males were significantly lower than those recorded for either control treatment across all morphological regions (BPA vs Control_solvent_: fins: *P* < 0.001, head: *P* < 0.001, and body: *P* < 0.001; BPA vs Control_H2O_: fins: *P* < 0.001, head: *P* < 0.001, and body: *P* < 0.001; Fig. 4). Male color scores did not differ between Control_solvent_ and Control_H2O_ males (Control_H2O_ vs Control_solvent_: fins: *P* = 0.84, head: *P* = 0.95, and body: *P* = 0.93; Fig. 4).

Comparison of summed (fins+body+head) pre-exposure (day 1) and post-exposure (day 15) color scores of *C. lutrensis* males exposed to BPA indicated that mean overall color intensity decreased by approximately 56% over the 14-day exposure period. Significant reductions were observed in all individual color components [fin coloration (paired *t*_15_ = 12.65, *P* < 0.001), head coloration (*t*_15_ = 14.47, *P* < 0.001), and body coloration (*t*_15_ = 9.05, *P* < 0.001)]. Color intensity also decreased within the *C. lutrensis* Control_solvent_ treatment [fins (*t*_11_ = 2.32, *P* = 0.04), head (*t*_11_ = 2.73, *P* = 0.02), and body (*t*_11_ = 4.52, *P* = 0.001)]. However, a mancova performed upon the mean final intensity scores of all color components (fins, head, body), with treatment specified as the independent factor and initial score values (prior to exposure) specified as covariates, confirmed that the extent of male color loss was significantly greater for BPA-treated males across all morphological regions [head (*F*_1,23_ = 19.66, *P* < 0.001), fins (*F*_1,23_ = 37.40, *P* < 0.001), and body (*F*_1,23_ = 24.48, *P* < 0.001)].

##### Cyprinella venusta

Consistent with expectations, comparison of post-trial (day 15) color scores for individual males across all trial series did not detect an effect of exposure treatment on caudal spot intensity (anova: *F*_2,56_ = 1.06; *P* = 0.34). Comparison of mean pre-exposure (day 1) and post-exposure (day 15) scores for males within the Control_solvent_ treatments indicated that all Control_solvent_ and BPA males retained maximal color during the exposure period (score = 3 for all males; statistical tests not conducted). Only one male demonstrated a reduction in the intensity of the caudal spot following exposure to BPA; accordingly, mean changes in the intensity of coloration over the exposure period were not significant (*t*_15_ = 1.00, *P* = 0.33).

#### Effects of exposure on male courtship

Exposure to BPA was not associated with significant overall reductions in the intensity of male courtship behavior. Mean total amounts of courtship performed by individual males toward both presented females [total time spent courting female *C. lutrensis* + total time spent courting female *C. venusta*)/2; number of courtship bouts directed toward female *C. lutrensis* + number of courtship bouts directed toward female *C. venusta*)/2] were similar among treatments (*C. lutrensis* males: *F*_4,110_ = 0.05; *P* = 0.99; *C. venusta* males: *F*_4,110_ = 0.92; *P* = 0.35). However, control male *C. lutrensis* generally courted females more aggressively than male *C. venusta*; in all treatments, *C. lutrensis* males spent more time engaged in courtship with females of both species than *C. venusta* [Control_solvent_: mean ± SEM = 126.03 ± 23.80 vs 48.45 ± 13.49 (*F*_1,38_ = 8.04, *P* = 0.007); Control_H2O_: mean ± SEM = 119.99 ± 23.79 vs 80.34 ± 19.97 (*F*_1,38_ = 1.63, *P* = 0.21); BPA: mean ± SEM = 132.79 ± 25.39 vs 51.51 ± 10.75 (*F*_1,36_ = 1.51, *P* = 0.23)]. *C. lutrensis* males in the BPA treatment also approached females more frequently than *C. venusta* males [Control_H2O_: mean ± SEM = 23.63 ± 3.75 vs 17.88 ± 3.71 (*F*_1,38_ = 1.19, *P* = 0.28); Control_solvent_: mean ± SEM = 24.48 ± 5.18 vs 15.65 ± 4.31 (*F*_1,38_ = 1.72, *P* = 0.20); BPA: mean ± SEM = 23.45 ± 4.30 vs 12.45 ± 2.45 (*F*_1,36_ = 4.95, *P* = 0.03)].

#### Effects of male trait variation on female response

Changes in female discrimination following BPA exposure were not strongly dependent on exposure-induced changes in male phenotypic trait expression. Analysis of covariance indicated that changes in the strengths of female responses to male *C. lutrensis* across treatments were not associated with variation in male *C. lutrensis* body coloration (treatment × color interaction: response to *C. lutrensis* males: *F*_2,100_ = 2.18, *P* = 0.12; Table S2; Fig. S1). We also did not find evidence that the relationships between male courtship intensity and the strengths of female response varied with respect to treatment (treatment × courtship interaction: response to *C. lutrensis* males: *F*_2,100_ = 2.63, *P* = 0.08, response to *C. venusta* males: *F*_2,106_ = 1.84, *P* = 0.17; Tables S2, S3; Figs. S2, S3).

## Discussion

Our data show that exposure to endocrine-disrupting chemicals (EDCs) can facilitate the breakdown of prezygotic reproductive isolation between closely related species. Levels of male courtship and female receptivity were similar across treatment groups, consistent with evidence that bisphenol A (BPA, generally considered to be a ‘weak’ xenoestrogen) does not inhibit overall willingness to spawn (Shioda and Wakabayashi 2000). However, the strength of prezygotic reproductive isolation between *Cyprinella* species exposed to BPA was significantly weaker than the strength of prezygotic isolation between *Cyprinella* species under control conditions. Qualitatively similar patterns of male and female responses within and between treatment groups suggest that exposure to BPA is associated with an increased propensity for males and females to approach and interact with heterospecific individuals in mixed-species breeding aggregations.

A reduction in the intensity of body color of male fish exposed to natural and synthetic estrogens has been well described in many species (e.g., Kristensen et al. 2005; Arellano-Aguilar and Garcia 2008), including *C. lutrensis* (McGree et al. 2010). We similarly observed uniform decreases in the intensities of *C. lutrensis* body and fin coloration in response to BPA, consistent with evidence that exposure to estrogenic EDCs, including BPA, is associated with decreased levels of androgens (Coe et al. 2008; Salierno and Kane 2009) responsible for regulating the expression of sexually dimorphic phenotypic traits in fish (Liley and Stacey 1983; Mayer et al. 2004). For example, in male carp, *Cyprinus carpio,* exposure to graded concentrations of BPA negatively correlated with plasma levels of testosterone and 11-ketotestosterone (Mandich et al. 2007). Bisphenol A also modulates the expression of ERα mRNA, aromatase, and gonodotropin subunit genes (gonadotropin-α, FSH-β, LH-β) associated with reproductive maturation and sexual dimorphism in killifish species (Rhee et al. 2010 and references therein). By contrast, BPA exposure did not affect the expression of the sexually monomorphic melanic caudal spot expressed by *C. venusta*. These results highlight possible functional differences between the information content in the two color signals – while both of these species-specific color patterns play a possible role in mate recognition, it is likely that only the carotenoid-based color in *C. lutrensis* is additionally used to facilitate intraspecific male discrimination.

We did not observe significant reductions in the courtship intensity of males treated with BPA, which contrasts with the results of other exposure studies involving natural and synthetic estrogens (17β-estradiol and 17α-ethinyl estradiol: Bayley et al. 1999; Bjerselius et al. 2001; Kristensen et al. 2005; Saaristo et al. 2010; McGree et al. 2010). For example, McGree et al. (2010) showed that both nuptial coloration and the frequencies of courtship displays were suppressed in *C. lutrensis* exposed to 17β-estradiol for 84 days. This difference could possibly be due to differences in exposure duration, but could also reflect the considerably weaker estrogenic potential of BPA compared to that of other natural and synthetic estrogens used in prior studies (Tabata et al. 2001).

Changes in female discrimination did not appear to be tightly linked to the expression of male phenotypic traits. Few other studies have explicitly examined how exposure to environmental hormones and hormone mimics influences female assessment strategies or mate choice. Arellano-Aguilar and Garcia (2008) found that female amarillo fish (*Girardinichthys multiradiatus*) exposed to an estrogenic insecticide discriminated against exposed, feminized conspecific males. Coe et al. (2008) showed that dominant zebrafish females (*Danio rerio*) are more likely to mate with subordinate conspecific males following exposure to 17α-ethinylestradiol. Additional experiments are needed to distinguish between the potential mechanisms underlying changes in female responses to male visual signals (e.g., unrecorded male variables that females may respond to) and to examine more specifically the role that changes in the expression of male signals may have in intraspecific mate choice and phenotypic evolution, as well as interspecific reproductive dynamics.

Our results contribute to a growing body of evidence demonstrating that the effects of human-mediated environmental alteration can extend well beyond individual-level reproductive success, with significant evolutionary consequences for populations and species (Hendry et al. 2008; Smith and Bernatchez 2008; Candolin and Wong 2012). The exposure of natural populations to estrogenic chemicals can lead to changes in communication that concomitantly change the strength and direction of sexual selection on phenotypic traits (Shenoy et al. 2010; van der Sluijs et al. 2010; Rosenthal et al. [Bibr b43]), potentially resulting in the loss of populations. Kidd et al. (2007), for example, showed that a population of fathead minnows (*Pimephales promelas*) collapsed owing to feminization of males and altered oogenesis in females resulting from chronic exposure to low concentrations (4.8–6.1 ng L^−1^) of the potent synthetic estrogen 17α-ethynylestradiol. Here, we show that exposure-induced changes in communication and assessment can increase the likelihood of hybridization between sympatric species. Our results indicate that the presence of EDCs in the environment can weaken sexual isolation between congeners and potentially lead to species decline either through the loss of reproductive effort or through the erosion of species boundaries. Hybridization is a contributing factor to widespread reductions in aquatic biodiversity (Miller et al. 1989), especially in areas that support highly diverse fish assemblages (Seehausen et al. 1997; Walters et al. 2008; Ward et al. 2012).

Our findings also suggest that EDCs in the environment could promote the establishment and spread of non-native species. Biological invasions are among the most significant threats to aquatic biodiversity worldwide (Dudgeon et al. 2006), and the likelihood and pace of biological invasions involving hybridization are inversely related to the strength of reproductive barriers between native and non-native species (Hall et al. 2006). Thus, by weakening barriers to hybridization, EDCs in the environment could further escalate loss of native aquatic biodiversity by accelerating the spread of invasive species.

## Data archiving statement

Data for this study are available at Dryad: doi:10.5061/dryad.th251

## Supporting Information

**Figure S1.** Scatterplots depicting the relationships between the mean strengths of female *C. lutrensis* and female *C. venusta* preferences for male *C. lutrensis* and corresponding male *C. lutrensis* body coloration (total intensity score: fins+head+body) in (A,B) Control_solvent_ trials; (C,D) Control_H2O_ trials and (E,F) BPA trials.

**Figure S2.** Scatterplots depicting the relationships between the mean strengths of female *C. lutrensis* and female *C. venusta* preferences for male *C. lutrensis* and corresponding male *C. lutrensis* PCA courtship score in (A,B) Control_solvent_ trials; (C,D) Control_H2O_ trials and (E,F) BPA trials.

**Figure S3.** Scatterplots depicting the relationships between the mean strengths of female *C. lutrensis* and female *C. venusta* preferences for male *C. venusta* and corresponding male *C. venusta* PCA courtship score in (A,B) Control_solvent_ trials; (C,D) Control_H2O_ trials and (E,F) BPA trials.

**Table S1.** Set-up of interspecific mate choice tests.

**Table S2.** Male visual traits and the strength of *Cyprinella lutrensis* and *C. venusta* female responses to male *C. lutrensis* in control (solvent, H_2_O) and BPA treatments (*F*_17,100_ = 18.18, *P* < 0.001).

**Table S3**. Male visual traits and the strength of *C. lutrensis* and *C. venusta* female responses to male *C. venusta* in control (solvent, H_2_O) and BPA treatments (*F*_11,106_ = 12.59, *P* < 0.001).

## References

[b1] Arellano-Aguilar O, Garcia C (2008). Exposure to pesticides impairs the expression of fish ornaments reducing the availability of attractive males. Proceedings of the Royal Society of London B.

[b2] Arukwe A (2001). Cellular and molecular responses to endocrine-modulators and the impact on fish reproduction. Marine Pollution Bulletin.

[b3] Bayley MJ, Nielsen JR, Baatrup E (1999). Guppy sexual behavior as an effect biomarker of estrogen mimics. Ecotoxicology and Environmental Safety.

[b4] Bjerselius R, Lundstedt-Enkel K, Olsen H, Mayer I, Dimberg K (2001). Male goldfish reproductive behavior and physiology are severely affected by exogenous exposure to 17-beta-estradiol. Aquatic Toxicology.

[b5] Blum MJ, Walters DM, Burkhead NM, Freeman BJ, Porter BA (2010). Reproductive isolation and the expansion of an invasive hybrid swarm. Biological Invasions.

[b6] Borg B (1994). Androgens in teleost fishes. Comparative Biochemistry and Physiology.

[b7] Broughton RE, Vedala KC, Crowl TM, Ritterhouse LL (2011). Current and historical hybridization with differential introgression among three species of cyprinid fishes (genus: *Cyprinella*. Genetica.

[b8] Candolin U, Wong BBM (2012). Behavioural Responses to a Changing World: Mechanisms and Consequences.

[b9] Candolin U, Salesto T, Evers M (2007). Changed environmental conditions weaken sexual selection in sticklebacks. Journal of Evolutionary Biology.

[b10] Casalini M, Agbali M, Reichard M, Konecná M, Bryjová A, Smith C (2009). Male dominance, female mate choice, and intersexual conflict in the rose bitterling (*Rhodeus ocellatus*. Evolution.

[b11] Clement TS, Grens KE, Fernald RD (2005). Female affiliative preference depends on reproductive state in the African cichlid fish, *Astatotilapia burtoni*. Behavioural Ecology.

[b12] Coe T, Hamilton P, Hodgson D, Paull G, Stevens J, Sumner K, Tyler C (2008). An environmental estrogen alters reproductive hierarchies, disrupting sexual selection in group spawning fish. Environmental Science and Technology.

[b13] Cousins IT, Staples CA, Klecka GM, Mackay D (2002). A multimedia assessment of the environmental fate of Bisphenol A. Human and Ecological Risk Assessment.

[b14] Crain DA, Eriksen M, Iguchi T, Jobling S, Laufer H, Leblanc GA, Guillette LJ (2007). An ecological assessment of bisphenol A: evidence from comparative biology. Reproductive Toxicology.

[b15] Cripe GM, Hemmer BL, Goodman LR, Fournie JW, Raimondo S, Vennari JC, Danner RL (2009). Multigeneration exposure of the estuarine sheepshead minnow (*Cyprinodon variegatus*) to 17beta- estradiol. I. Organism-level effects over 3 generations. Environmental Toxicology and Chemistry.

[b16] Dudgeon D, Arthington AH, Gessner M, Kawabata OZI, Knowler DJ, Leveque C, Naiman RJ (2006). Freshwater biodiversity: importance, threats, status and conservation challenges. Biological Reviews.

[b17] Fisher HS, Wong B, Rosenthal GG (2006). Alteration of the chemical environment disrupts communication in a freshwater fish. Proceedings of the Royal Society of London B.

[b18] Gabor CR, Grober MS (2010). A potential role of male and female androgen in species recognition in a unisexual–bisexual mating complex. Hormones and Behavior.

[b19] Gale WF (1986). Indeterminate fecundity and spawning behavior of captive Red Shiners—fractional, crevice spawners. Transactions of the American Fisheries Society.

[b20] Hall RJ, Hastings A, Ayres DR (2006). Explaining the explosion: modeling a hybrid invasion. Proceedings of the Royal Society of London B.

[b21] Hendry AP, Farrugia TJ, Kinnison MT (2008). Human influences on rates of phenotypic change in wild animal populations. Molecular Ecology.

[b22] Hubbs C, Strawn K (1956). Interfertility between two sympatric fishes, *Notropis lutrensis* and *Notropis venustus*. Evolution.

[b23] Jobling S, Tyler CR (2003). Endocrine disruption in wild freshwater fish. Pure and Applied Chemistry.

[b24] Kidd KA, Blanchfield PJ, Mills KH, Palace VP, Evans RE, Lazorchak JM, Flick RW (2007). Collapse of a fish population after exposure to a synthetic estrogen. Proceedings of the National Academy of Science of the United States of America.

[b25] Kolpin DW, Furlong ET, Meyer MT, Thurman EM, Zaugg EM, Barber LB, Buxton HT (2002). Pharmaceuticals, hormones, and other organic wastewater contaminants in U.S. streams, 1999–2000 a national reconnaissance. Environmental Science and Technology.

[b26] Kozak GM, Head ML, Boughman JW (2011). Sexual imprinting on ecologically divergent traits leads to sexual isolation in sticklebacks. Proceedings Royal Society B.

[b27] Kristensen T, Baatrup E, Bayley M (2005). 17-alpha-ethinyl estradiol reduces the competitive reproductive fitness of the male guppy (*Poecilia reticulata*. Biology of Reproduction.

[b28] Liley NR, Stacey NE, Hoar WS, Randall DJ, Donaldson EM (1983). Hormones, pheromones, and reproductive behavior in fish. Fish Physiology, Vol. 9: Reproduction, Part B: Behavior and Fertility Control.

[b29] Lynch KS, Crews D, Ryan MJ, Wilczynski W (2006). Hormonal state influences aspects of female mate choice in the Túngara Frog (*Physalaemus pustulosus*. Hormones and Behavior.

[b30] Mandich A, Bottero S, Benfenati E, Cevasco A, Erratico C, Maggioni S, Massari A (2007). In vivo exposure of carp to graded concentrations of bisphenol A. General and Comparative Endocrinology.

[b31] Mayer I, Borg B, Páll M (2004). Hormonal control of male reproductive behavior in fishes: a stickleback perspective. Behaviour.

[b32] McGree MM, Winkelman DL, Vieira NKM, Vajda AM (2010). Reproductive failure of the red shiner (*Cyprinella lutrensis*) after exposure to an exogenous estrogen. Canadian Journal of Fisheries and Aquatic Science.

[b33] Mendelson TC (2003). Sexual isolation evolves faster than hybrid inviability in a diverse and sexually dimorphic genus of fish (Percidae: *Etheostoma*. Evolution.

[b34] Miller RR, Williams JD, Williams JE (1989). Extinction of North American fishes during the past century. Fisheries.

[b35] Mills LJ, Chichester C (2005). Review of evidence: are endocrine-disrupting chemicals in the aquatic environment impacting fish populations?. Science of the Total Environment.

[b36] Minckley WL (1972). Notes on the spawning behavior of Red Shiner, introduced into Burro Creek, Arizona. Southwestern Naturalist.

[b37] Page LM, Burr BM (1991). A field guide to freshwater fishes.

[b38] Page LM, Smith RL (1970). Recent range adjustments and hybridization of *Notropis lutrensis* and *Notropis spilopterus* in Illinois. Transactions of the Illinois Academy of Science.

[b39] Partridge C, Boettcher A, Jones AG (2010). Short-term exposure to a synthetic estrogen disrupts mating dynamics in a pipefish. Hormones and Behavior.

[b40] Ptacek M (2000). The role of mating preferences in shaping interspecific divergence in mating signals in vertebrates. Behavioural Processes.

[b41] Ramsey ME, Wong RY, Cummings ME (2011). Estradiol, reproductive cycle and preference behavior in a northern swordtail. General and Comparative Endocrinology.

[b42] Rhee JS, Kim R-O, Seo JS, Kang HS, Park CB, Soyano K, Lee J (2010). Bisphenol A modulates expression of gonadotropin subunit genes in the hermaphroditic fish, *Kryptolebias marmoratus*. Comparative Biochemistry and Physiology C.

[b43] Rosenthal GG, Stuart-Fox D, Candolin U, Wong BBM (2009a). Environmental disturbance and animal communication. Behavioural responses to a changing world: mechanisms and consequences.

[b44] Saaristo M, Craft JA, Lehtonen KK, Lindström K (2012). Sand goby (*Pomatoschistus minutus*) males exposed to an endocrine disrupting chemical fail in nest and mate competition. Hormones and Behavior.

[b45] Saaristo M, Craft JA, Lehtonen KK, Bjork H, Lindström K (2009b). Disruption of sexual selection in sand gobies (*Pomatoschistus minutus*) by 17α-ethinylestradiol, an endocrine disruptor. Hormones and Behavior.

[b46] Saaristo M, Craft JA, Lehtonen KK, Lindström K (2010). Exposure to 17-alpha ethinyl estradiol impairs courtship and aggressive behavior of male sand gobies (*Pomatoschistus minutus*. Chemosphere.

[b47] Salierno JD, Kane AS (2009). 17-alpha-ethinyl estradiol alters reproductive behaviors, circulating hormones and sexual morphology in male fathead minnows (*Pimephales promelas*. Environmental Toxicology and Chemistry.

[b48] Seehausen O, Witte JJM, van Alphen F (1997). Cichlid fish diversity threatened by eutrophication that curbs sexual selection. Science.

[b49] Seehausen O, Takimoto G, Roy D, Jokela J (2008). Speciation reversal and biodiversity dynamics with hybridization in changing environments. Molecular Ecology.

[b50] Shenoy K, Crowley PH (2010). Endocrine disruption of male mating signals: ecological and evolutionary implications. Functional Ecology.

[b51] Shioda T, Wakabayashi M (2000). Effect of certain chemicals on the reproduction of medaka (*Oryzias latipes*. Chemosphere.

[b52] van der Sluijs I, Gray SM, Amorim MCP, Barber I, Candolin U, Hendry A, Krahe R (2010). Communication in troubled waters: responses of fish communication systems to changing environments. Evolutionary Ecology.

[b53] Smith TB, Bernatchez L (2008). Evolutionary change in human-altered environments. Molecular Ecology.

[b54] Sohoni P, Tyler CR, Hurd K, Caunter J, Hetheridge M, Williams T, Woods C (2001). Reproductive effects of long-term exposure to bisphenol A in the fathead minnow (*Pimephales promelas*. Environmental Science and Technology.

[b55] Stelkens RB, Seehausen O (2009). Phenotypic divergence but not genetic distance predicts assortative mating among species of a cichlid fish radiation. Journal of Evolutionary Biology.

[b56] Tabata A, Kashiwada S, Ohnishi Y, Ishikawa H, Miyamoto N, Magara Y (2001). Estrogenic influences of estradiol-17β, p-nonylphenol and bisphenol A on Japanese medaka (*Oryzias latipes*) at detected environmental concentrations. Water Science and Technology.

[b57] Taylor EB, Boughman JW, Groenenboom M, Sniatynksi M, Schluter D, Gow J (2006). Speciation in reverse: morphological and genetic evidence of a collapse of a stickleback species pair (*Gasterosteus*. Molecular Ecology.

[b58] Thompson RR, George K, Dempsey J, Walton JC (2004). Visual sex discrimination in goldfish: seasonal, sexual, and androgenic influences. Hormones and Behavior.

[b59] Walters DM, Blum MJ, Rashleigh B, Freeman BJ, Porter BA, Burkhead NM (2008). Red shiner invasion and hybridization with blacktail shiner in the upper Coosa River, USA. Biological Invasions.

[b60] Ward JL, McLennan D (2009). Female mate choice based upon complex visual cues in the brook stickleback, *Culaea inconstans*. Behavioral Ecology.

[b61] Ward JL, Blum MJ, Walters DM, Freeman BJ, Porter BA, Burkhead NM (2012). Discordant introgression in a rapidly expanding hybrid swarm. Evolutionary Applications.

