# Supplementary material for: Exposure to an environmental estrogen breaks down sexual isolation between native and invasive species
Source: Evol Appl. 2012 Jul 10;5(8):901–12. doi: 10.1111/j.1752-4571.2012.00283.x (PMC3552407; doi:10.1111/j.1752-4571.2012.00283.x)

Supporting Information

Supporting Information Table 1: Set-up of interspecific mate choice tests.

**Trial series**	***n***	**Exposure regime/Focal individuals**			
		***C. venusta* female**	***C. venusta* male**	***C. lutrensis* male**	***C. lutrensis* female**
1	20	$Control_{H2O}$	$Control_{H2O}$	$Control_{H2O}$	…
2	20	$Control_{solvent}$	$Control_{solvent}$	$Control_{solvent}$	…
3	19	BPA	BPA	BPA	…
4	20	…	$Control_{H2O}$	$Control_{H2O}$	$Control_{H2O}$
5	20	…	$Control_{solvent}$	$Control_{solvent}$	$Control_{solvent}$
6	19	…	BPA	BPA	BPA

I. *Preliminary analyses*

At the start of the experiment, *C. lutrensis* and *C. venusta* allocated to $Control_{solvent}$, $Control_{H2O}$ and BPA treatment groups were comparable within species and sex classes. Pre-exposure (Day 1) intensities of male body coloration did not differ across treatments [ANOVA: *C. lutrensis* males*:* head ($F_{1,26}$ = 0.19, P = 0.67), fins ($F_{1,26}$ = 1.11, P = 0.30), body ($F_{1,26}$ = 1.48, P = 0.24); *C. venusta* males: caudal spot = 3 for all males in both groups (statistical tests not performed)]. With the exception of $Control_{solvent}$ male *C. venusta* exhibiting larger standard lengths than conspecific males in the other treatments (ANOVA: $F_{2,56}$ = 6.41, P = 0.003), *C. lutrensis* and *C. venusta* individuals did not differ in size (standard length) within species and sex classes (*C. lutrensis* females: $F_{2,56}$ = 0.47, P = 0.63; *C. lutrensis* males: $F_{2,56}$ = 0.35, P = 0.70; *C. venusta* females: $F_{2,56}$ = 1.56, P = 0.22). We did not find evidence that the behavior of either sex varied as a function of standard length within any treatment (Pearson correlations: all P > 0.05).

Preliminary screening indicated that neither the solvent, nor the exposure set-up affected the behavior of experimental males and females. Comparisons conducted separately for each sex within each species indicated that male and female *C. lutrensis* and *C. venusta* in both control treatments ($Control_{solvent}$, $Control_{H2O}$) responded to conspecific or heterospecific mates in a similar manner [MANOVA: females (*C. lutrensis*: $F_{4,35} = 1.77$, $P = 0.16$; *C. venusta*: $F_{4,35} = 0.59$, $P = 0.68$); males (*C. lutrensis*: $F_{4,35} = 0.03$, $P = 0.99$; *C. venusta*: $F_{4,35} = 0.75$, $P = 0.56$)]. We compared data from all three treatments in final analyses.

II. *Female responses to male phenotypic traits*

Male color and courtship intensity were not correlated across treatments in response to females of either species (Pearson correlations: $P > 0.05$). Variables were normally distributed (1-sample Kolmogorov-Smirnov test: all $P > 0.05$) and satisfied the assumption of variance homogeneity (Levene's test: $P > 0.05$).

Supporting Information Table 2: Male visual traits and the strength of *Cyprinella lutrensis* and *C. venusta* female responses to male *C. lutrensis* in control (solvent, $H2O$) and BPA treatments ($F_{17,100}$ =18.18, $P < 0.001$).

**Factor**	**df**	***F***	***P***
Treatment	2,100	1.06	0.35
Female species	1,100	6.17	**0.02**
Color	1,100	1.87	0.18
Courtship	1,100	126.36	**< 0.01**
Female x Courtship	1,100	4.55	**0.04**
Female x Color	1,100	0.68	0.41
Treatment x Courtship	2,100	2.63	0.08
Treatment x Color	2,100	2.18	0.12
Treatment x Female species	2,100	1.50	0.23
Treatment x Female x Courtship	2,100	2.01	0.14
Treatment x Female x Color	2,100	0.51	0.60

Supporting Information Table 3: Male visual traits and the strength of *C. lutrensis* and *C. venusta* female responses to male *C. venusta* in control (solvent, $H2O$) and BPA treatments ($F_{11,106}$ =12.59, $P < 0.001$).

**Factor**	**df**	***F***	***P***
Treatment	2,106	0.05	0.95
Female species	1,106	24.84	**<0.01**
Courtship	1,106	50.33	**<0.01**
Female x Courtship	1,106	17.16	**<0.01**
Treatment x Courtship	2,106	1.84	0.17
Treatment x Female species	2,106	6.81	**<0.01**
Treatment x Female x Courtship	2,106	0.72	0.49

Supporting Information Figure 1: Scatterplots depicting the relationships between the mean strengths of female *C. lutrensis* and female *C. venusta* preferences for male *C. lutrensis* and corresponding male *C. lutrensis* body coloration (total intensity score: fins+head+body) in (A,B) $Control_{solvent}$ trials; (C,D) $Control_{H2O}$ trials and (E,F) BPA trials.

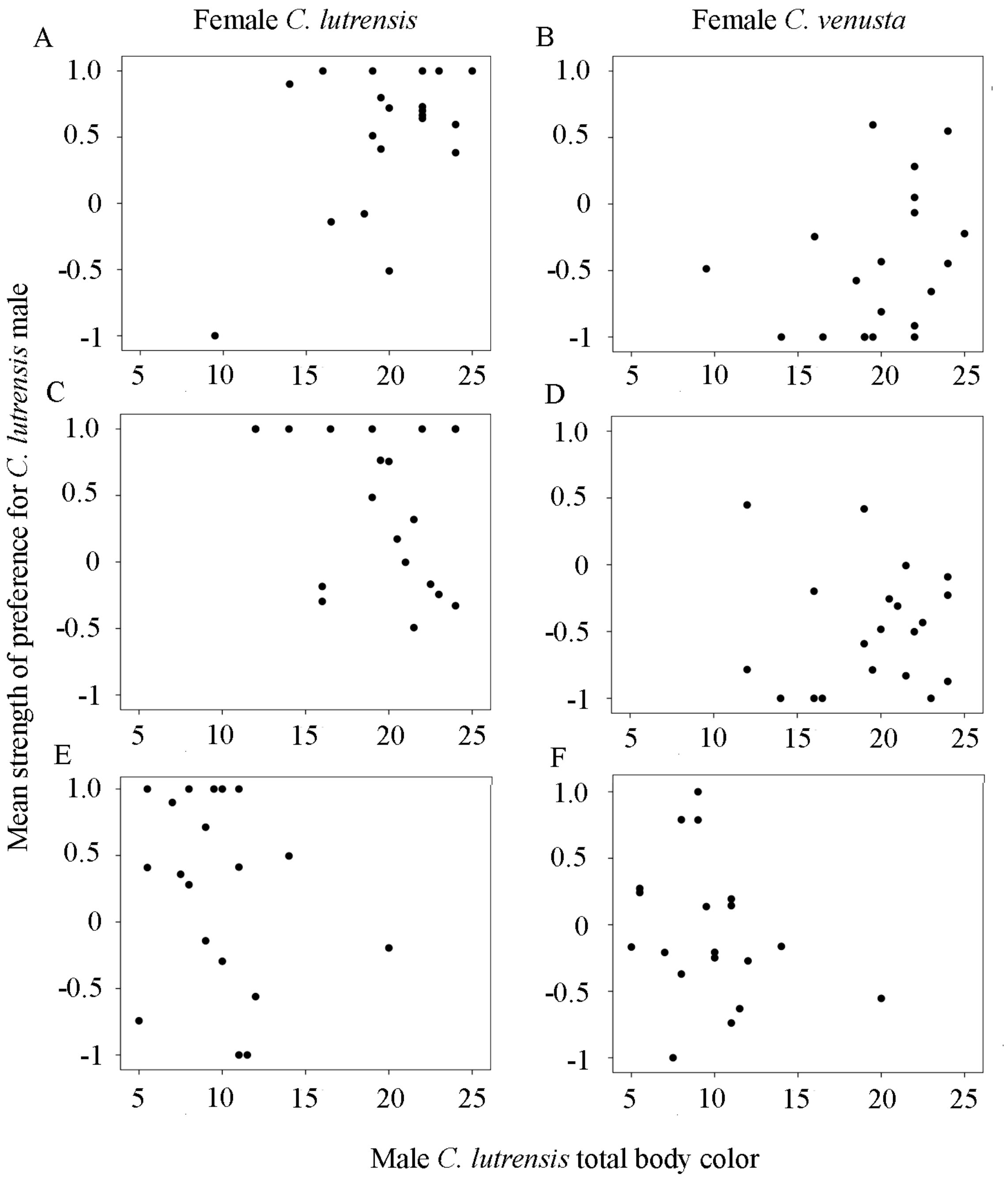

Supporting Information Figure 2: Scatterplots depicting the relationships between the mean strengths of female *C. lutrensis* and female *C. venusta* preferences for male *C. lutrensis* and corresponding male *C. lutrensis* PCA courtship score in (A,B) Control$_{solvent}$ trials; (C,D) Control$_{H2O}$ trials and (E,F) BPA trials.

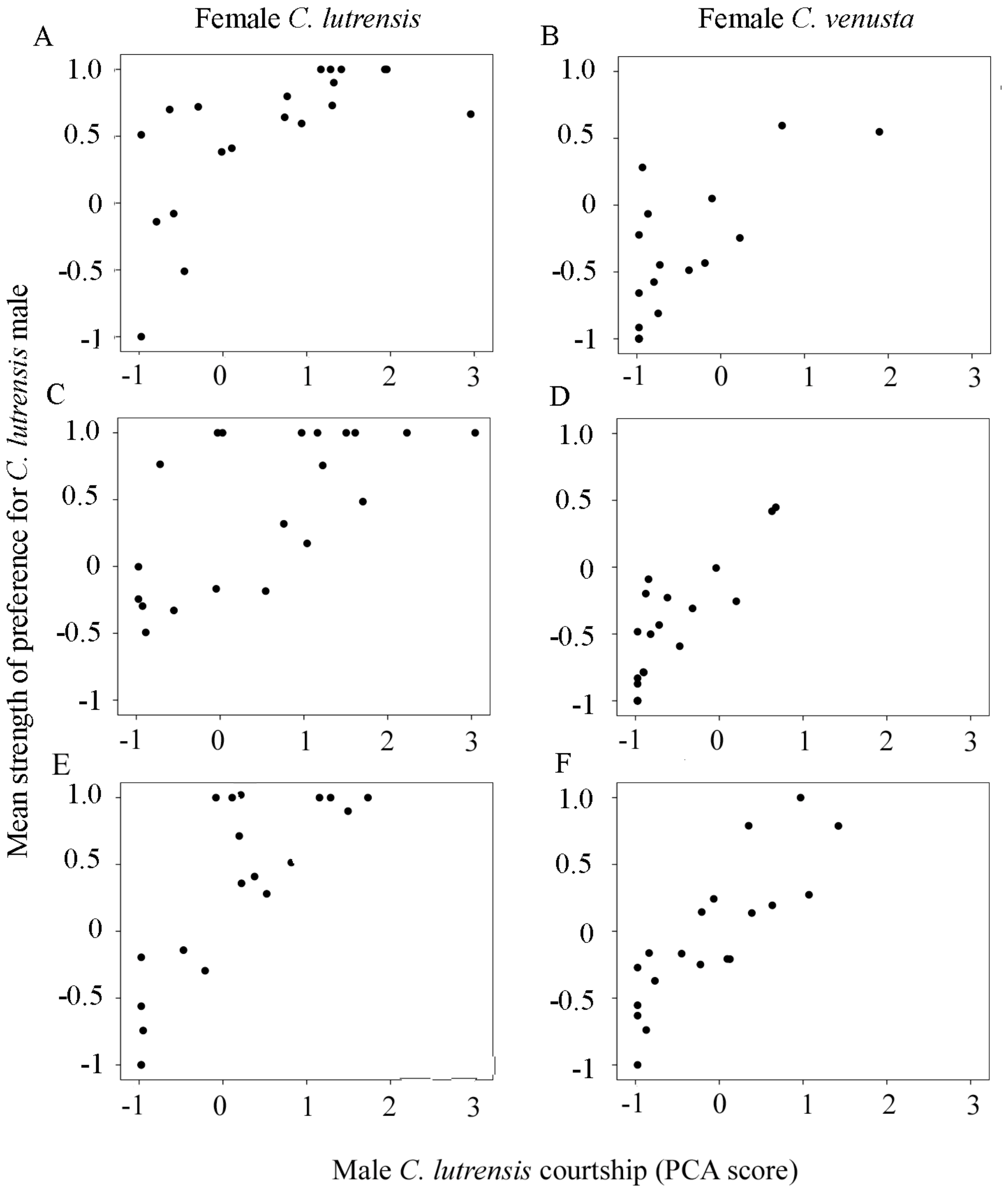

Supporting Information Figure 3: Scatterplots depicting the relationships between the mean strengths of female *C. lutrensis* and female *C. venusta* preferences for male *C. venusta* and corresponding male *C. venusta* PCA courtship score in (A,B) $\text{Control}_{\text{solvent}}$ trials; (C,D) $\text{Control}_{\text{H2O}}$ trials and (E,F) BPA trials.

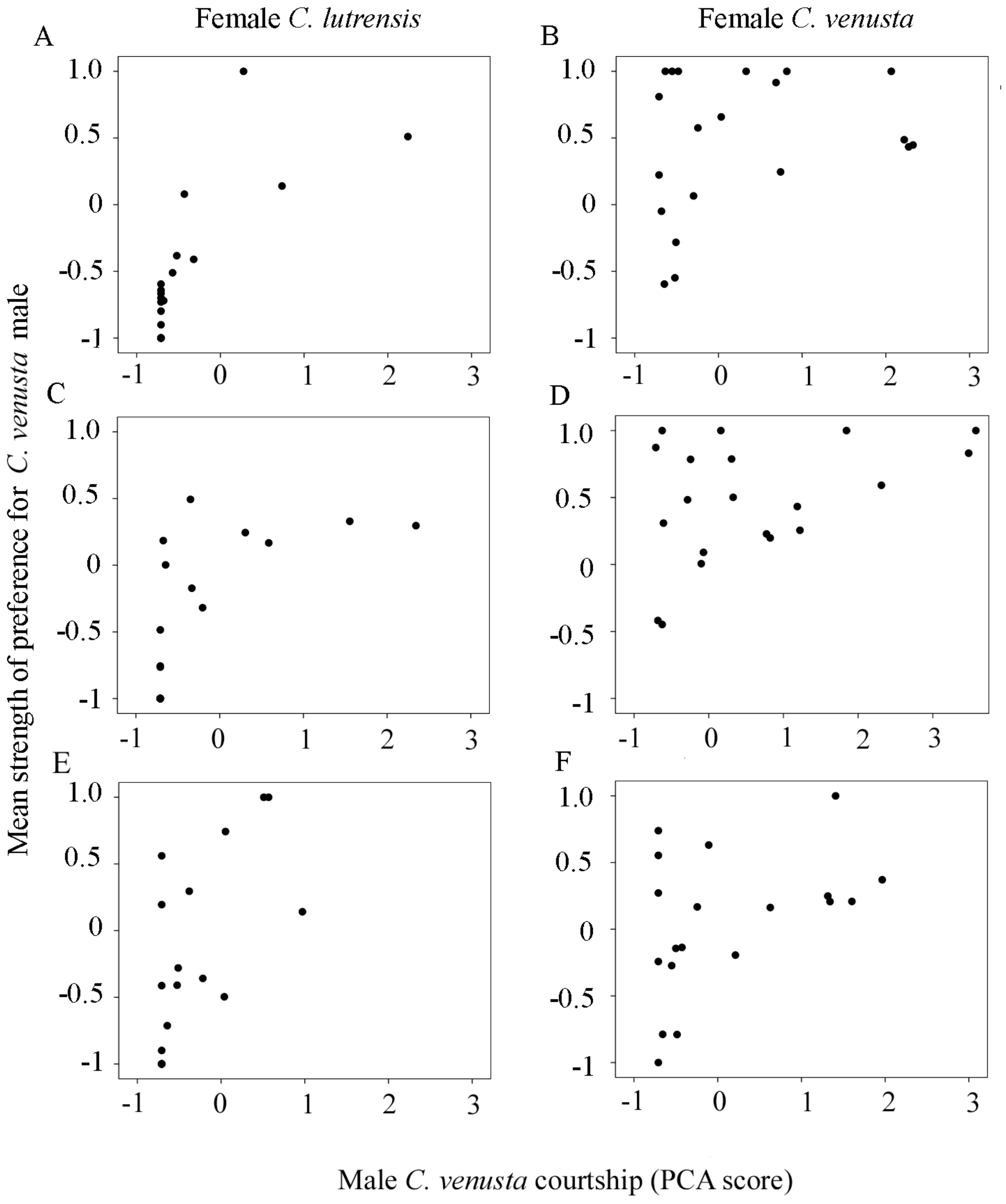

Supplement: Supplementary file 1 [file eva0005-0901-SD1.pdf]
