# Supplementary figures and images for: Exposure to an environmental estrogen breaks down sexual isolation between native and invasive species
Source: Evol Appl. 2012 Jul 10;5(8):901–12. doi: 10.1111/j.1752-4571.2012.00283.x (PMC3552407; doi:10.1111/j.1752-4571.2012.00283.x)

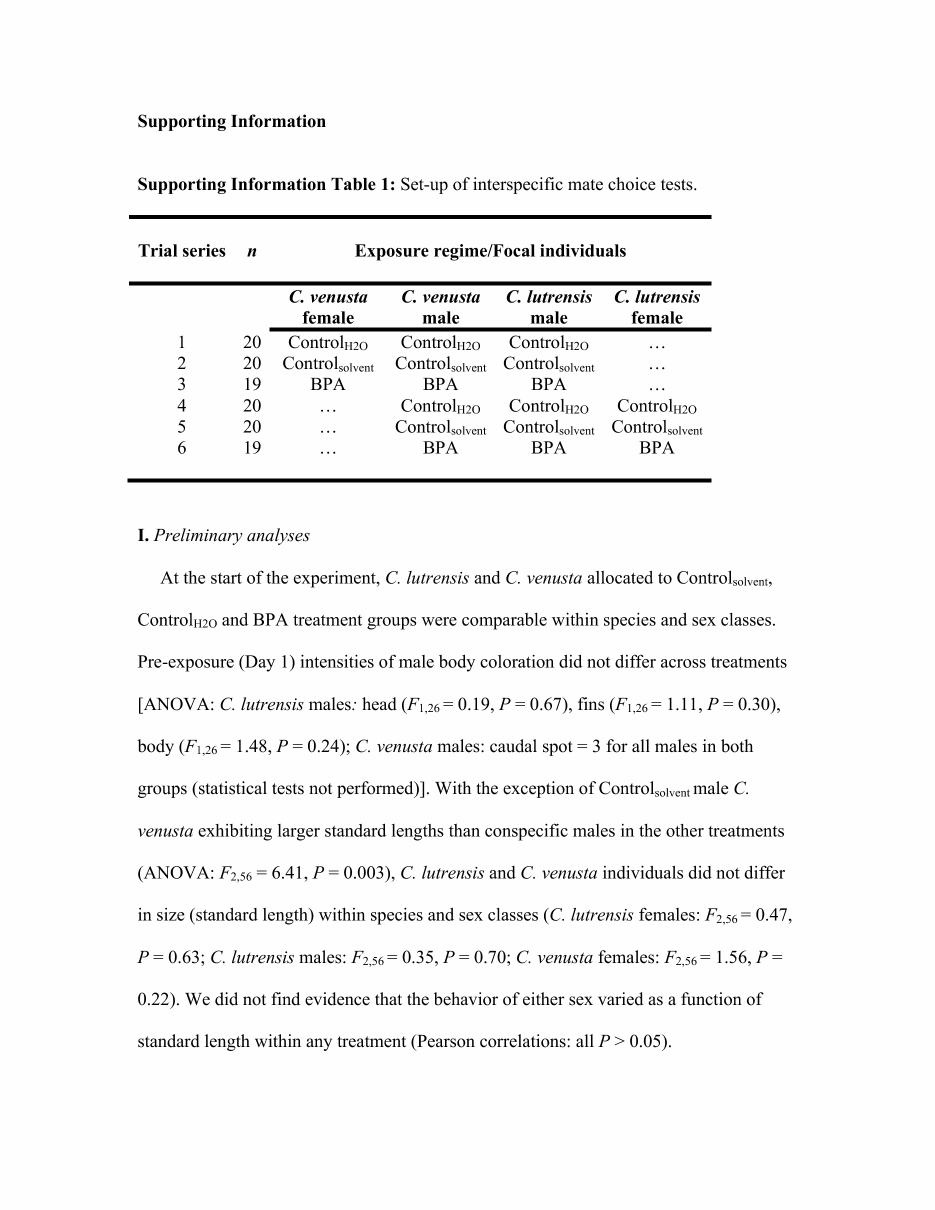

Supplement: Supplementary file 2 [file eva0005-0901-SD2.png]
